# Molecular epidemiology and virulence profiles of MDR and XDR *Pseudomonas aeruginosa* from combat-related wounds in Ukraine

**DOI:** 10.1016/j.nmni.2026.101880

**Published:** 2026-10-30

**Authors:** Oana Palasca, Caroline Hvidberg Schwartz, Rasmus Kristensen, Iryna Koshova, Bohdan Murashevych, Trine Rolighed Thomsen, Frederik Boëtius Hertz, Thomas Bjarnsholt, Tim Holm Jakobsen

**Affiliations:** a Costerton Biofilm Center, Department of Immunology and Microbiology, University of Copenhagen, Copenhagen, Denmark; b Department of Microbiology, Virology, Immunology, Epidemiology, and Biomedical Physics and Informatics, Dnipro State Medical University, Dnipro, Ukraine; c Department of Biochemistry and Medical Chemistry, Dnipro State Medical University, Dnipro, Ukraine; d Danish Technological Institute, Aarhus University, Aarhus, Denmark; e Department of Chemistry and Bioscience, Aalborg University, Aalborg, Denmark; f Department of Clinical Microbiology, Rigshospitalet, Copenhagen, Denmark

**Keywords:** *Pseudomonas aeruginosa*, Whole-genome sequencing, Antimicrobial resistance, Combat-related wounds, Molecular epidemiology, Carbapenemase

## Abstract

**Background:**

Combat-related injuries sustained during the ongoing conflict in Ukraine have been associated with infections caused by multidrug-resistant Gram-negative bacteria, raising concerns about treatment options and cross-border transmission to receiving healthcare systems. *Pseudomonas aeruginosa* is among the most clinically significant pathogens in this setting. This study aimed to define the molecular epidemiology of *P. aeruginosa* from combat-related wounds in Ukraine and to link genomic resistance and virulence determinants to antimicrobial susceptibility and virulence phenotypes.

**Methods:**

*P. aeruginosa* isolates from distinct wound patients (n = 13, Dec 2024–Jun 2025) underwent whole-genome sequencing, SNP-based phylogenetic analysis, and screening of acquired resistance genes and chromosomal resistance-associated mutations. Antimicrobial susceptibility testing followed EUCAST, and carbapenemase production was assessed phenotypically. Virulence profiling included *exoU/exoS* carriage, 3-oxo-C12-HSL and C4-HSL production, and pyocyanin and rhamnolipid production.

**Results:**

Ten sequence types were identified, including ST235, ST244, ST773, and ST1047, indicating high clonal diversity rather than a single outbreak lineage. All isolates were non-susceptible to ciprofloxacin, ceftazidime, piperacillin–tazobactam, and aztreonam; 5/13 were non-susceptible to ceftolozane–tazobactam and 4/13 were meropenem-resistant. Carbapenemase-positive isolates carried bla_IMP-1_ (3/13) or bla_NDM-1_ (1/13). Resistance-associated chromosomal variation affected porins, efflux regulators, and β-lactam response pathways. Virulence profiles were heterogeneous, with mutually exclusive *exoU* (7/13) or *exoS* (6/13) carriage and variable pyocyanin and rhamnolipid production.

**Conclusions:**

These data provide genomic and phenotypic characterisations of combat-wound *P. aeruginosa* from Ukraine, highlighting substantial diversity and frequent multidrug-resistant phenotypes and supporting lineage-resolved genomic surveillance in conflict-associated care pathways.

## Highlights

• Ten sequence types among 13 isolates demonstrated substantial clonal diversity.
• A high frequency of MDR/XDR phenotypes was observed among the 13 isolates.
• Meropenem non-susceptibility was restricted to isolates carrying blaIMP-1 or blaNDM-1.
• Chromosomal variants in *oprD*, AmpC-associated pathways, and efflux regulators were common.

## 1 Introduction

*Pseudomonas aeruginosa* is a major opportunistic pathogen causing hospital-acquired pneumonia, bloodstream infections, urinary tract infections, and infections of burns and traumatic wounds [1]. It is particularly associated with healthcare-associated infections in critically ill and immunocompromised patients and is a frequent cause of infection in combat-related wounds [2], [3]. Clinical management is challenging because of intrinsic antimicrobial resistance, metabolic versatility, and ability to persist in damaged tissue [4], [5].

Resistance in *P. aeruginosa* is often multifactorial, involving acquired carbapenemases and other β-lactamases together with chromosomal mutations affecting porins, efflux regulation, and β-lactam response pathways [4], [6]. Horizontal gene transfer further contributes to the dissemination of antimicrobial resistance determinants, which may be carried by integrons and other mobile genetic elements, including plasmids, transposons, and integrative and conjugative elements [7]. Consequently, infections may be multidrug resistant (MDR) or extensively drug resistant (XDR), requiring last-line or combination therapies with limited treatment options [8], [9]. In the 2024 WHO Bacterial Priority Pathogens List, carbapenem-resistant *P. aeruginosa* was classified as a high-priority pathogen [10].

Combat-related injuries create conditions that further complicate infection management and control. Gram-negative bacteria have emerged as a major clinical and public health concern in war-wounded patients [11], [12], [13], and recent reports from Ukraine and receiving healthcare systems have documented highly resistant organisms among evacuated patients, raising concerns regarding both treatment and cross-border transmission [14], [15], [16].

The global epidemiology of *P. aeruginosa* is shaped by internationally disseminated high-risk and epidemic lineages that contribute disproportionately to antimicrobial resistance and human infection, with some lineages showing adaptation to particular host or clinical populations [17], [18]. ST235 is among the most prevalent high-risk clones worldwide and is frequently associated with carbapenemase acquisition and severe disease [19]. Other lineages, including ST111, ST175, ST244, ST773, and ST1047, are also increasingly recognized in clinical and healthcare-associated settings, with heterogeneous resistance and virulence profiles [20], [21], [22]. ST1047 has recently been linked to conflict- and displacement-associated healthcare networks and carbapenemase acquisition, underscoring the importance of lineage-resolved surveillance in settings with cross-border patient movement [23], [24]. MDR pathogens in patients evacuated from Ukraine have been reported in European healthcare settings, resulting in difficult-to-treat infections [16], [25], [26], [27], [28].

At the molecular level, antimicrobial resistance and virulence in *P. aeruginosa* are driven by a combination of acquired genes and chromosomally encoded determinants. Carbapenem resistance is frequently mediated by metallo-β-lactamases (MBLs), including bla_NDM-1_, bla_IMP-1_, and bla_VIM_, while aminoglycoside resistance may be enhanced by 16S rRNA methyltransferases such as *rmtB* [29], [30], [31]. Virulence potential is strongly influenced by type III secretion system effectors, particularly ExoU and ExoS. The *exoU* and *exoS* genes are strongly associated with distinct major phylogenetic lineages of *P. aeruginosa* [32], [33], with *exoU*-positive strains frequently associated with increased cytotoxicity and acute disease, whereas *exoS* predominates in the other major lineage and is common among strains associated with chronic infection [33], [34].

Detailed molecular epidemiological data on *P. aeruginosa* from combat-related wounds in Ukraine have been reported previously, including studies documenting MDR and XDR isolates and high-risk clones in conflict-associated settings [35], [36]. However, studies that simultaneously integrate clonal lineage assignment, multi-tool resistance profiling, virulence gene content, and quantitative virulence phenotyping remain scarce for this population. We therefore combined whole-genome sequencing, antimicrobial susceptibility testing, and virulence phenotyping to characterize *P. aeruginosa* isolated from combat-related wounds in Ukraine.

## 2 Methods

### 2.1 Bacterial strains and culture conditions

A total of thirteen clinical *P. aeruginosa* isolates were obtained from combat-related wounds from thirteen different patients (Supplementary Table 1). Isolates originated from multiple anatomical sites. Cultures were grown in LB broth at 37 °C while shaking at 185 rpm. Isolates were stored in LB supplemented with 20% glycerol at −80 °C.

### 2.2 MALDI-TOF MS

Species identity was confirmed for all isolates by matrix-assisted laser desorption/ionization-time of flight mass spectrometry (MALDI-TOF MS) (Bruker Daltonik) using the MBT Compass software (v. 4.1); all isolates scored ≥2.0, indicating high-confidence identification.

### 2.3 Whole genome sequencing (WGS)

Genomic DNA was extracted from *P. aeruginosa* isolates in duplicates using the DNeasy Blood and Tissue Kit (Qiagen, Germany), following the manufacturer's protocol. Sequencing libraries were prepared with the Nextera XT DNA Library Prep Kit (Illumina, USA) and sequenced on an Illumina MiSeq platform, generating 150-bp paired-end reads. An average of ∼8,000,000 read pairs per isolate (range: ∼2,200,000–11,700,000) was obtained.

### 2.4 Genome assembly, resistance, and virulence predictions

De novo assemblies were generated using Shovill (v1.4.2). Assembly quality was assessed based on the total assembled genome length. Given the expected genome size of *P. aeruginosa* (∼6.3–7.0 Mb), assemblies with total lengths exceeding 8 Mb were considered indicative of potential misassembly or contamination. Although flagged individually, their retention as sequencing duplicates remained informative: cross-replicate confirmation of gene calls ensured that any artefacts specific to a single assembly were discarded, as described below. The oversized assemblies likely arose from residual low-level DNA contamination introduced during library preparation or from non-specific amplification in the Nextera XT protocol, which is a known limitation of this approach for low-biomass or mixed-input samples. The duplicate-confirmation strategy was adopted specifically to mitigate this risk: a gene was considered present only if detected in both replicates by at least one of the three annotation tools, thereby filtering assembly-specific artefacts regardless of their origin. This conservative approach reduces sensitivity marginally but substantially increases specificity for gene calls, ensuring that downstream genotype–phenotype associations are based on robustly confirmed genomic content rather than assembly-specific noise.

### 2.5 Multi-locus sequence typing

Multi-locus sequence typing (MLST) [37] was determined using the MLST 2.0 service provided by the Center for Genomic Epidemiology.

### 2.6 Comparative phylogenomics with publicly available genomes

To place our isolates in a global phylogenetic context, we reconstructed a phylogeny including a representative subset of publicly available *P. aeruginosa* genomes. All assemblies annotated as *P. aeruginosa* (complete genomes, scaffolds, and contigs) were downloaded from NCBI on 05/03/2026. Assemblies were filtered for quality by retaining genomes with total sequence length between 5.8 and 7.2 Mb, ≤500 contigs, and contig N50 ≥ 20,000 bp. Only GenBank assemblies (accessions starting with “GCA_”) were included. STs (sequence types) were assigned to all genomes using the mlst command-line tool. To reduce sampling bias while preserving population structure, genomes were subsampled proportionally to ST abundance. For STs represented by more than five assemblies, up to 0.02% of available genomes (minimum of one per ST) were retained. Additional genomes were randomly sampled from the remaining pool of rare STs to obtain a final dataset of 1500 public genomes. These were combined with isolates from this study and selected reference genomes (PAO1, GCA_000006765.1; PA14, GCA_000014625.1; LESB58, GCA_000026645.1), and outgroup taxa used for tree rooting (*P. paraeruginosa* PA7, GCA_000017205.1; *P. paraeruginosa*, GCA_003025345.2; *P. stutzeri*, GCA_019704535.1; *P. putida*, GCA_000007565.2; *Azotobacter vinelandii*, GCA_000021045.1).

Protein-coding genes for all genomes were predicted using Prodigal (v2.6.3) [38]. Forty universal single-copy marker genes were identified using fetchMGs (v2.0.0) [39] in gene mode. Genomes lacking one or more of the 40 marker genes were excluded from downstream analysis (n = 20). Protein sequences were aligned for each gene using MAFFT (v7.490) [40], back-translated to nucleotide sequences, and concatenated into a super matrix. The alignment was trimmed using trimAl (v1.4.rev22) [41] with a gap threshold of 0.1. A maximum-likelihood phylogeny was inferred using IQ-TREE (v2.3.5) [42] with ModelFinder [43] for substitution model selection. Branch support was assessed using 1000 ultrafast bootstrap and 1000 SH-aLRT replicates (-B 1000 -alrt 1000), with additional options to improve robustness (-nm 5000 -bnni) [44], [45]. The tree was rooted on the outgroup taxa for visualization. For our isolates, final analyses were performed using one representative assembly per isolate.

### 2.7 Resistance and virulence predictions

Antimicrobial resistance genes were identified using Resistance Gene Identifier (RGI) (v6.0.5) via the CARD web interface (v4.0.1) [46], as well as ResFinder [47] (staramr v0.12.1) and AMRFinderPlus [48] (v3.12.8) executed via the Galaxy platform [49]. For RGI, only “strict” or “perfect” hits were retained. For ResFinder, a minimum identity threshold of 90% and minimum coverage of 60% were applied, while for AMRFinderPlus, minimum identity and coverage thresholds of 90% and 80%, respectively, were used. Results from the three tools were combined by taking the union of all detected genes. A gene was considered present only if detected in both sequencing duplicates by at least one of the applied tools. Virulence-associated genes were detected using ABRicate (v1.0.1) with the VFDB [50] database in Galaxy, applying minimum identity and coverage thresholds of 90% and 80%, respectively. Virulence genes were only considered present if detected in both duplicate assemblies, thereby ensuring robust gene calls and minimizing the impact of assembly-specific artefacts.

### 2.8 SNP calling, phylogenetics, and recombination analysis

Whole-genome sequencing reads were analysed using the BacDist pipeline [51]. Reads were mapped to the *P. aeruginosa* PAO1 reference genome (GenBank: GCA_000006765.1) using Snippy (v4.6.0) with default high-stringency parameters. High-quality variants were combined into a multi-sample VCF, and SNP-only alignments were generated for downstream analyses. Maximum-likelihood phylogenetic trees were inferred using RAxML (v8.2.11) [52] under the GTRCAT model. Homologous recombination was assessed using ClonalFrameML [53], and SNPs located within predicted recombination regions were excluded from phylogenetic analyses. Final analyses were performed using one representative assembly per isolate.

### 2.9 Variant calling for genotype-phenotype analysis

Single-sample variant calls generated by Snippy were used without recombination filtering, retaining all high-quality single-nucleotide variants identified relative to the PAO1 reference genome. Non-synonymous variants in resistance- and virulence-associated genes were extracted and analysed in relation to antimicrobial susceptibility and phenotypic measurements.

### 2.10 Antimicrobial susceptibility testing

Clinical isolates were grown on LB plates overnight to single colonies. A colony was resuspended in 2 mL 0.9% saline solution, and turbidity was adjusted to a 0.5 McFarland standard. With a cotton swab, the suspension was transferred to a Mueller Hinton (MH) broth II agar plate (Millipore, Sigma Aldrich) ensuring the whole plate was covered. Discs of selected antibiotics were placed on the surface of the plate, and the plates were incubated at 37 °C for 18 h. Inhibition zones were measured, and isolates were categorized as susceptible, intermediate, or resistant according to the European Committee on Antimicrobial Susceptibility Testing (EUCAST) guidelines (v 15.0, 2025). For MDR/XDR classification, both intermediate (I) and resistant (R) susceptibility categories were considered non-susceptible, in accordance with the Magiorakos et al. definition [54].

Antibiotics tested were ciprofloxacin (5 μg), sulfamethoxazole + trimethoprim (25 μg), meropenem (10 μg), piperacillin + tazobactam (36 μg), gentamicin (10 μg), ceftazidime (10 μg), tobramycin (10 μg), aztreonam (30 μg), and ceftolozane + tazobactam (40 μg) (Oxoid Ltd., England).

### 2.11 Minimum inhibitory concentration of colistin

The minimum inhibitory concentration (MIC) of colistin was determined using broth microdilution technique according to EUCAST guidelines. Briefly, overnight cultures grown in LB were adjusted to OD_600_ = 0.1 and diluted 1:200 in MH broth II. Bacterial suspensions were distributed into Nunc 96-well polypropylene plates (Thermo Fisher Scientific, USA) containing serial dilutions of colistin sulphate salt (Sigma Aldrich) and incubated at 37 °C for 18 h. *P. aeruginosa* PAO1 served as the quality control strain.

### 2.12 Carbapenemase production assay

Carbapenemase activity was evaluated using the modified carbapenem inactivation method (mCIM) and the EDTA-modified CIM (eCIM) according to Clinical & Laboratory Standards Institute (CLSI) guidelines. *P. aeruginosa* isolates showing phenotypic resistance to meropenem were tested. *E. coli* ATCC 25922 served as the indicator, and a serine carbapenemase-positive *K. pneumoniae* isolate was used as a positive control. Test isolates and reference strains were cultured on LB agar plates and incubated at 37 °C overnight. A 1 μL inoculum was resuspended in 2 mL tryptic soy broth (TSB) for mCIM. For eCIM, a second 1 μL inoculum was suspended in TSB containing 5 mM EDTA (applied only to isolates tested positive by mCIM). Meropenem discs (10 μg) were added to each suspension and incubated at 37 °C for 4 h.

MH broth II agar plates were inoculated with a 0.5 McFarland suspension of *E. coli* ATCC 25922. The incubated meropenem discs were transferred to the indicator plates and incubated at 37 °C for 18 h mCIM results were interpreted as positive (6-15 mm inhibition zone), intermediate (16-18 mm), or negative (≥19 mm). eCIM results were considered positive if the inhibition zone increased by ≥ 5 mm compared with mCIM, indicating MBL production, and negative if zone size < 4 mm.

### 2.13 Quorum sensing

Overnight cultures grown in LB broth were adjusted to an OD_600_ of 0.2 and incubated for 22 h. Cultures were then centrifuged at 10,000 x g for 10 min, and the supernatants were sterile filtered through 0.22 μm filters (Sigma Aldrich). Production of the QS signal molecules N-(3-oxododecanoyl)-L-homoserine lactone (3-oxo-C12-HSL) and N-butanoyl-L-homoserine lactone (C4-HSL), associated with the Las and Rhl QS systems, respectively was assessed using the GFP-based *E. coli* reporter strains MH155 (3-oxo-C12-HSL) [55] and MH205 (C4-HSL) [56] as previously described [57]. MH155 and MH205 carry AHL-responsive transcriptional reporter fusions to an unstable GFP reporter, *gfp*(ASV), allowing fluorescence to be used as a measure of AHL activity (Supplementary Table 1).

Monitor strains were grown on LB plates overnight, resuspended in B-minimal media, supplemented with 10% A-10, 0.5% (wt/vol) casamino acid, and 0.5% (wt/vol) glucose, and adjusted to OD_600_ of 2. Autoinducers were extracted from 1 mL culture supernatant of clinical isolates, PAO1, and *ΔQSI* [58] using acidified ethyl acetate (Sigma Aldrich) by two sequential extractions (1 mL solvent and centrifugation at 10,000 x g for 60 s). Extracts (1.6 mL) were evaporated and resuspended in 150 μL methanol (Sigma Aldrich). Aliquots (2 μL for 3-oxo-C12-HSL, 10 μL for C4-HSL) were dispensed into wells and solvent was evaporated prior to the addition of 100 μL monitor culture. GFP fluorescence was measured for 18 h using a Wallac 1420 VICTOR^2^ plate reader (PerkinElmer™, USA) with excitation at 485 nm and emission at 535 nm.

### 2.14 Rhamnolipid assay

To quantify the rhamnolipid production of the isolates, a modified version of [59] were used. A total of 300 μL culture supernatants, including supernatants from PAO1 and *ΔQSI*, were mixed with 1 mL ethyl acetate and centrifuged at 10,000 x g for 1 min. The organic phase was transferred to a new tube, and the extraction was repeated. The combined organic phase (1.8 mL) was evaporated and resuspended in 300 μL MilliQ. A reference dilution series of L-rhamnose monohydrate (Sigma) was produced. 100 μL extract was mixed with 100 μL 1.6% orcinol (Aldrich) and 800 μL 60% sulfuric acid (Carl Roth, Germany). This was repeated for the rhamnose dilution series. The samples were incubated at 80 °C for 30 min, then cooled on ice and transferred to a flat-bottomed microtiter plate for quantification in a Biotek Synergy 4 Multi-detection microplate reader (Agilent Technologies, USA), measuring the absorbance at 420 nm. Values were multiplied by a factor of 2.5 to convert the unit from rhamnose to rhamnolipid (mg/mL) [60].

### 2.15 Pyocyanin assay

2 mL supernatant and 1.2 mL chloroform (Sigma Aldrich) were mixed and centrifuged at 10,000 rpm for 10 min. 800 μL of the bottom layer was mixed with 400 μL 0.2 M HCl (Sigma Aldrich) and centrifuged at 10,000 rpm for 2 min before distributing the extract to a flat bottom 96-well plate. Absorbance was measured at 520 nm using a Biotek Synergy 4 Multi-detection microplate reader. Pyocyanin concentration (μg/mL) was calculated by multiplying measurements with 17.072 and 0.4 [61].

### 2.16 Statistical analysis

Phenotypic measurements were compared with PAO1 using one-way ANOVA followed by Dunnett's multiple-comparisons test. A *P* value < 0.05 was considered statistically significant. Experiments were performed in three independent replicates unless otherwise stated.

## 3 Results

### 3.1 Isolate collection, sequencing quality, and phylogenetic structure

Thirteen *P. aeruginosa* clinical isolates were collected from combat-related wounds in Ukraine between December 2024 and June 2025, with each isolate originating from a distinct patient (Fig. 1A). WGS was performed for all isolates, and technical duplicates were generated for selected isolates to assess analytical robustness. De novo assemblies generally fell within the expected genome size range; however, four assemblies exceeded 8 Mb and were considered atypical. These atypical assemblies originated from different isolates, and for each isolate, at least one corresponding assembly fell within the expected size range. Duplicate assemblies consistently clustered together in both SNP-based and marker gene phylogenies, confirming technical reproducibility.

**Fig. 1:**
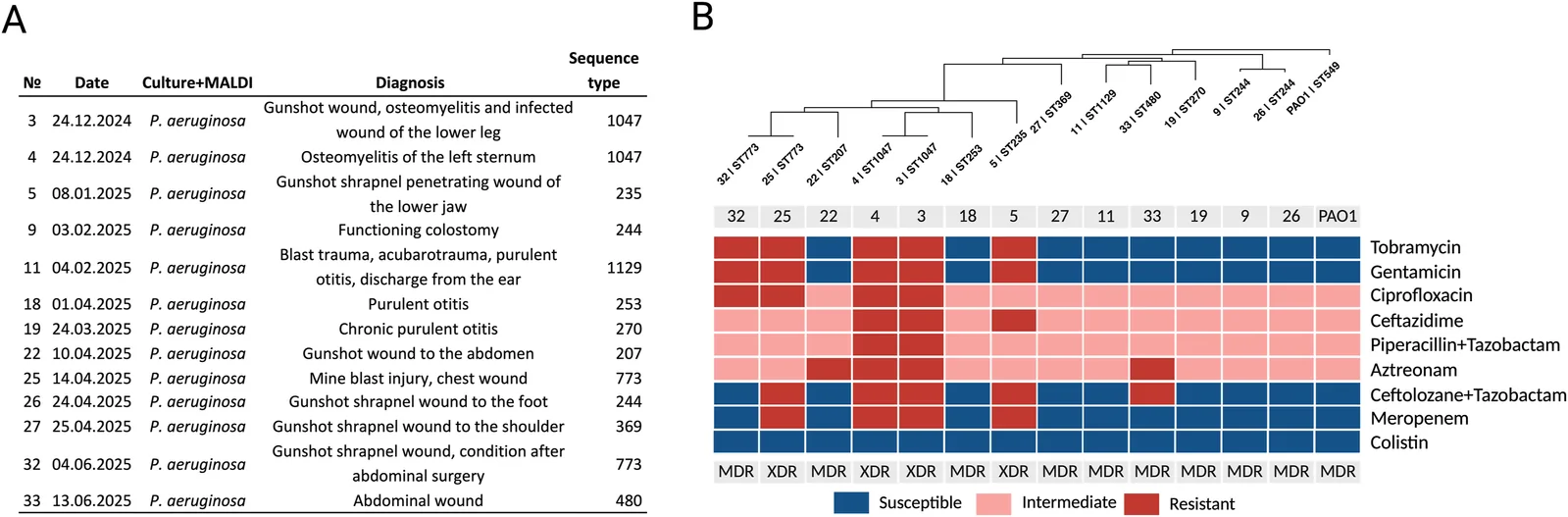
**Collection overview and antimicrobial susceptibility of combat-related wounds in Ukraine.** A. Overview of the 13 Ukrainian isolates, including isolate number, collection date, species identification, clinical diagnosis, and sequence type. B. Heatmap of phenotypic antimicrobial susceptibility profiles for selected antipseudomonal antibiotics. Susceptibility categories are shown as susceptible (S), intermediate (I), or resistant (R) according to EUCAST criteria. Isolates are ordered according to the phylogeny, allowing comparison of susceptibility patterns across sequence types and clades.

Variant calling showed a high concordance across technical duplicates, with approximately 98% of nonsynonymous variants shared, supporting the robustness of the SNP-based analyses. Recombination-aware analysis using ClonalFrameML inferred identical tree topology and only minor branch-length adjustments relative to the maximum-likelihood phylogeny indicating limited impact of homologous recombination on the phylogenetic signal.

Phenotypic antimicrobial susceptibility testing revealed high levels of resistance to multiple antipseudomonal antibiotics (Fig. 1B). Reduced susceptibility (intermediate or resistant) to ciprofloxacin, ceftazidime, piperacillin–tazobactam, and aztreonam was observed in all isolates. Reduced susceptibility to ceftolozane–tazobactam was detected in 5/13 isolates, and to meropenem in 4/13 isolates. All meropenem-non-susceptible isolates tested positive for carbapenemase production. In total, four isolates were categorized as extensively drug-resistant (XDR), and the remaining isolates as MDR according to previously accepted guidelines [54].

### 3.2 High clonal diversity with multiple internationally disseminated sequence types

MLST analysis revealed substantial clonal diversity among the 13 *P. aeruginosa* isolates, identifying a total of 10 distinct STs. Several internationally disseminated lineages were detected, including ST235, ST244, ST253, ST773, and ST1047. Among these, ST1047, ST244, and ST773 were each represented by two isolates, while the remaining STs were represented by single isolates (Fig. 1A). Comparison with publicly available genomes showed that several of the sequence types identified in our collection have been reported across multiple geographic regions, placing the Ukrainian isolates within both widely distributed and less frequently represented clonal backgrounds (Fig. 2A). A comparison with previously published *P. aeruginosa* genomes from Ukraine (n = 117) further showed that several of the STs identified here have already been reported in the country, supporting their continued presence in this setting (Fig. 2B). Notably, ST1047 was the most frequently represented ST among the publicly available Ukrainian genomes included in this comparison, in contrast to the global pattern, where ST235, ST253, and ST244 were more frequently represented (Fig. 2A and B). This over-representation in publicly available Ukrainian genomes is consistent with prior reports linking ST1047 to conflict- and displacement-associated healthcare networks, though the small size of the present collection precludes conclusions about local expansion or clonal dominance.

**Fig. 2:**
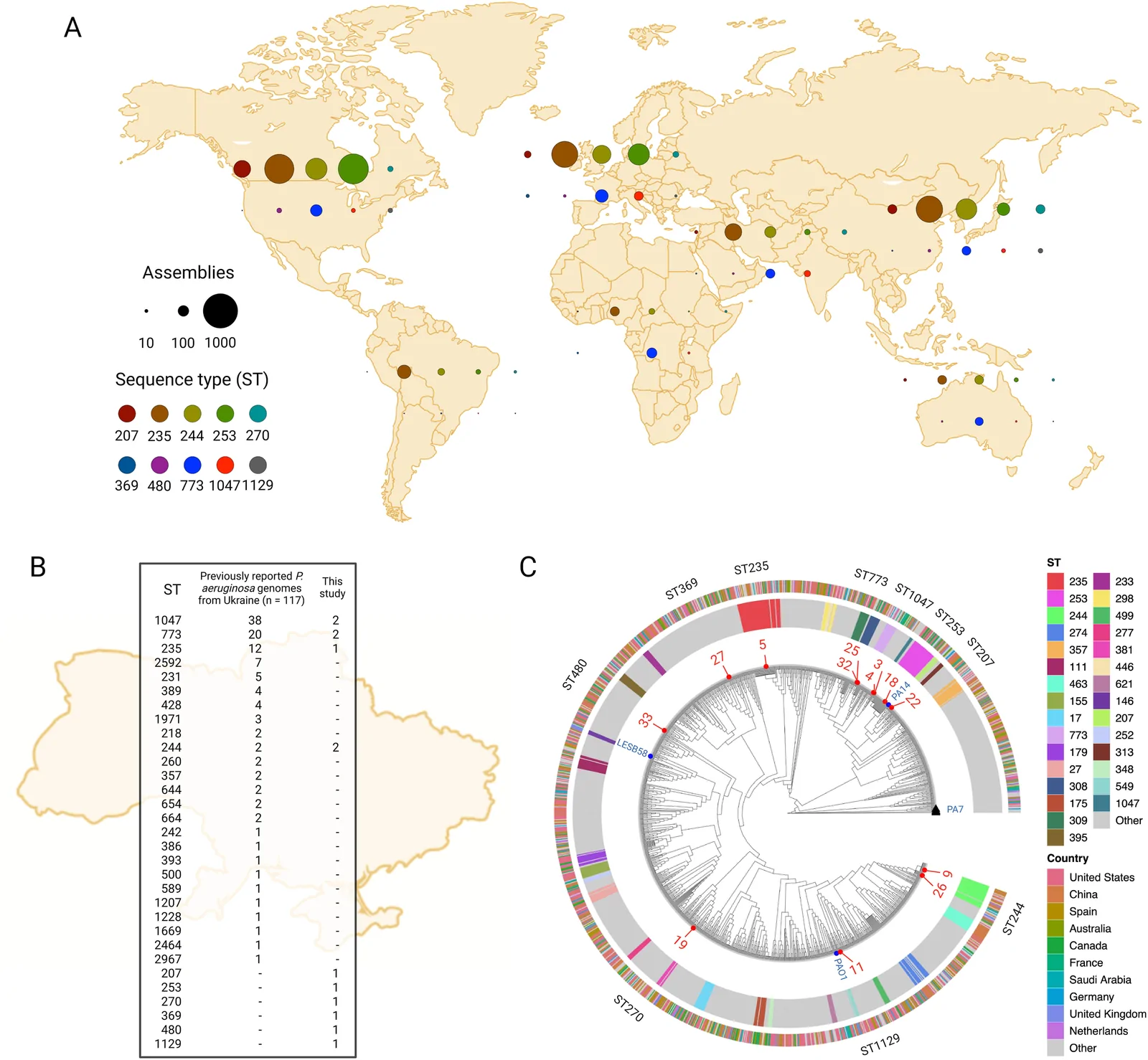
**Global and national sequence type distribution and population structure of***P. aeruginosa***isolates from combat-related wounds in Ukraine.** A. Global distribution of published genomes belonging to the sequence types identified in this study. Circle size indicates the number of assemblies, and colours denote sequence type. B. Sequence types (STs) previously reported from Ukraine in published genomes (n = 117) compared with those identified in this study. C. Global phylogeny of *P. aeruginosa* isolates based on 40 universal single-copy marker genes. The phylogeny includes 1500 publicly available genomes subsampled to reflect ST abundance, together with the 13 isolates from this study, selected reference genomes (PAO1, PA14, LESB58 and PA7) and three outgroup genomes. Genomes lacking one or more marker genes were excluded. The tree is rooted using the outgroup genomes. Circular annotations denote key isolate attributes. The inner ring shows the 30 most abundant MLST STs, illustrating clustering by ST. The outer ring represents the 10 most frequent countries of origin, highlighting limited geographic structuring. The remaining categories are grouped as “Other”. Isolates from this study are highlighted in red, and reference genomes are shown in blue. Outgroup genomes are indicated by black triangles.

To place these isolates in a global context, we compared them with a representative set of 1500 publicly available *P. aeruginosa* genomes using a phylogeny based on 40 universal single-copy marker genes (Fig. 2C). Consistent with the MLST results, the study isolates were distributed across the global *P. aeruginosa* phylogeny rather than forming a single clonal cluster. Isolates belonging to ST1047, ST773, ST253, and ST207 clustered within the PA14-like major lineage. Consistent with previous population-genomic studies, this lineage is strongly enriched for *exoU*-positive isolates, although *exoU* carriage is not an absolute marker of phylogroup membership [32], [33]. The ST235 isolate was also *exoU*-positive but showed variable placement across IQ-TREE runs, likely reflecting limited support for its precise position in the present marker-gene phylogeny. The remaining isolates were distributed across multiple clades and did not cluster tightly with one another. Together, these findings indicate that combat-related wound infections in Ukraine are caused by genetically diverse *P. aeruginosa* sequence types spanning both globally disseminated high-risk clones and more heterogeneous backgrounds, rather than a single expanding clone.

### 3.3 Genomic determinants of antimicrobial resistance

Genomic analysis revealed a heterogeneous distribution of antimicrobial resistance determinants across the isolate collection, broadly concordant with the observed phenotypic resistance patterns (Fig. 3). In addition to the intrinsic resistance background of *P. aeruginosa*, resistance-associated chromosomal variation affected porins and antibiotic response pathways, efflux regulatory network and quinolone target genes. Acquired carbapenemase genes were detected in all carbapenemase-positive isolates. bla_IMP-1_ was identified in three isolates (3, 4, 5) belonging to ST1047 and ST235 backgrounds, whereas bla_NDM-1_ was detected in isolate 25 (ST773). Besides carbapenemases, several additional acquired β-lactamase genes were detected with isolate-specific carriage patterns: bla_CTX-M-15_ (32), bla_OXA-1_ (22), bla_OXA-10_ (3, 4), bla_OXA-50_ (11), bla_OXA-395_ (11, 22, 25, 32), bla_OXA-486_ (19), bla_OXA-488_ (3, 4, 5, 18), bla_OXA-494_ (9, 26), bla_OXA-847_ (9, 26), bla_OXA-904_ (33), bla_OXA-1028_ (19), bla_OXA-1276_ (33), bla_TEM-1b_ (5) and bla_TEM-219_ (22), together with multiple chromosomal bla_PDC_ allelic variants. This expanded β-lactamase repertoire highlights the marked diversity of β-lactamase resistance determinants across the collection (Supplementary Table S2–S6).

**Fig. 3:**
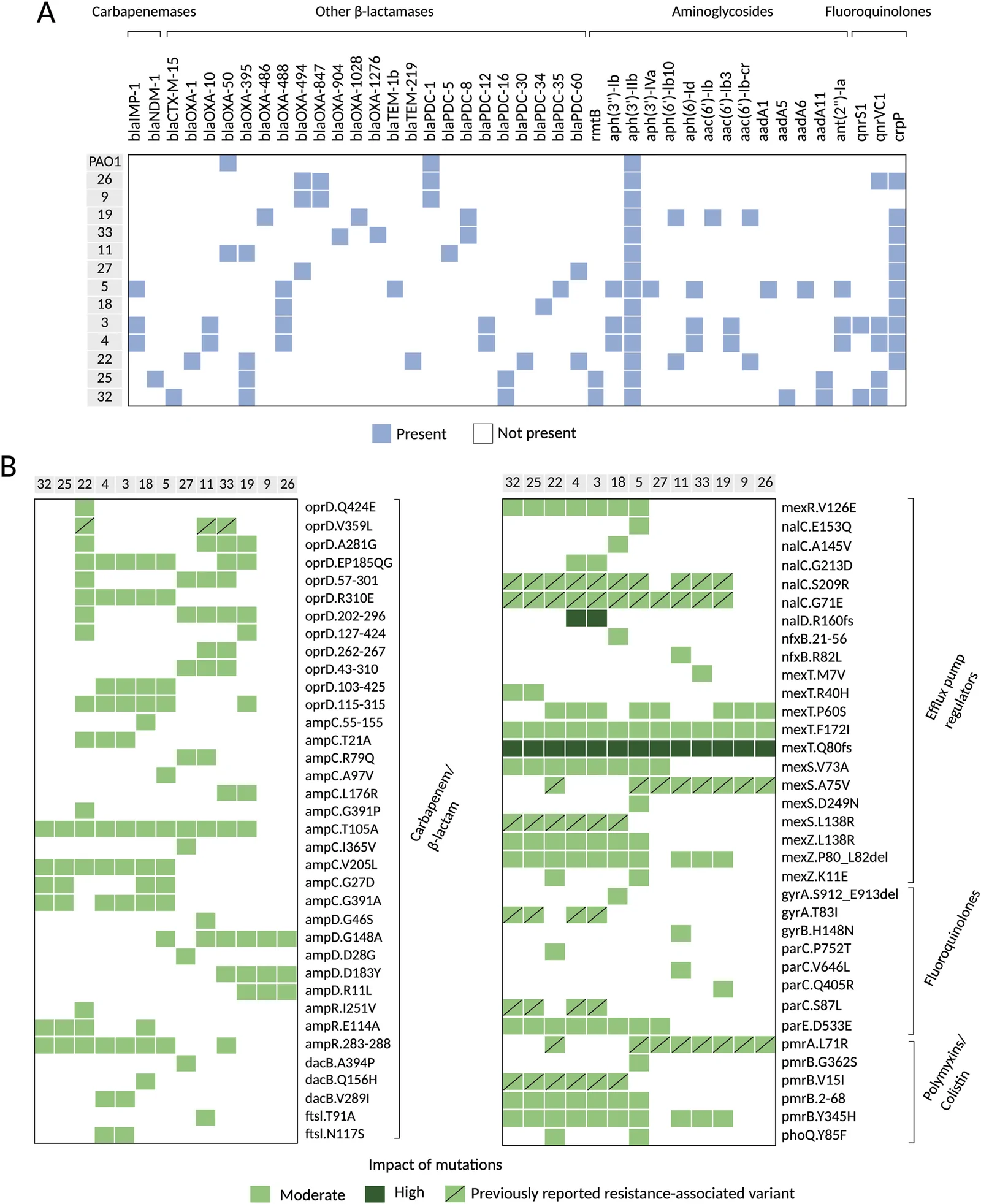
**Acquired resistance genes and resistance-associated chromosomal mutations in*****P. aeruginosa*****isolates from combat-related wounds in Ukraine.** A. Presence/absence matrix of selected acquired antimicrobial resistance genes across the 13 *P. aeruginosa* isolates. Genes are grouped by functional class, including carbapenemases, other β-lactamases, aminoglycoside resistance determinants, and fluoroquinolone-associated genes. Filled squares indicate gene presence and white indicates absence. Gene presence was defined according to tool-specific thresholds and required detection in both sequencing duplicates by at least one of the applied tools (RGI, ResFinder, or AMRFinderPlus). B. Heatmap of moderate- and high-impact chromosomal mutations in selected resistance-associated loci. Mutations are grouped by functional category, including carbapenem/β-lactam resistance determinants (*oprD*, *ampC* and AmpC-regulatory genes), efflux pump regulators (e.g., *mexR*, *nalC*, *nalD*, *nfxB*, *mexT*), fluoroquinolone resistance genes (*gyrA*, *gyrB, parC*, *parE*), and polymyxin/colistin-associated regulatory systems (*pmrA/pmrB*, *phoQ*). Colour indicates predicted mutation impact (moderate or high). Hatched boxes indicate mutations also reported in CARD and/or AMRFinderPlus. Isolates are ordered consistently with the SNP-based phylogeny.

Genes associated with aminoglycoside resistance were detected in all isolates, including the 16S rRNA methyltransferase gene *rmtB*, which was identified in two isolates (25, 32) belonging to ST773. Fluoroquinolone resistance–associated mutations were detected in the quinolone resistance–determining regions of *gyrA* and *parC* in isolates exhibiting ciprofloxacin resistance. High-level ciprofloxacin resistance was strongly associated with the canonical *gyrA* Thr83Ile substitution [62], which was present in all fully ciprofloxacin-resistant isolates (3, 4, 25, 32). Additional substitutions were observed in *parC*, supporting stepwise accumulation of quinolone resistance determinants (Fig. 3A).

All isolates harboured β-lactam resistance determinants, including genes associated with AmpC β-lactamase regulation (Fig. 3B). Multiple nonsynonymous variants in the carbapenem porin OprD were detected across the isolate collection. The functional consequences of individual variants were not experimentally determined in this study. The OprD Val359Leu substitution [63] was detected in a subset of isolates (11, 22, 33). Nonsynonymous variants in *ampD*, a regulator of AmpC β-lactamase expression, were identified across the isolate collection. Additional mutations were observed in *dacB* (3, 4, 18, 27), a gene previously implicated in β-lactam resistance through AmpC derepression, with distinct substitutions detected across multiple isolates [64]. Several isolates carried substitutions in colistin-associated regulatory genes (*phoQ* Y85F; *pmrA* L71R; *pmrB* V15I, Y345H, and G362S). However, all isolates remained colistin-susceptible (MIC 0.5–1 mg/L), indicating that these variants were not sufficient to confer a resistant phenotype in these strains. In addition, a recurrent missense mutation in the MexAB–OprM efflux repressor MexR (Val126Glu) was identified in multiple resistant isolates [65]. Variants were also detected in other efflux regulatory genes, including *nalC****,*** *nalD****,*** *nfxB***,** and *mexT***.** Their individual contributions to the observed resistance phenotypes were not experimentally determined (Supplementary Table S7–S9).

### 3.4 Heterogeneous distribution of virulence-associated genes

Genomic analysis revealed a heterogeneous distribution of virulence-associated genes across the *P. aeruginosa* isolate collection (Fig. 4). Fig. 4A displays genes with variable presence across isolates, whereas core virulence determinants conserved in all isolates are reported in Supplementary Table S10+S11. Variation was observed in genes associated with the type III secretion system (T3SS), most notably the effector genes *exoU* and *exoS*. The cytotoxic effector gene *exoU* was detected in seven isolates belonging to multiple sequence types, while *exoS* was present in the other six isolates. Iron acquisition systems were broadly distributed, with pyochelin biosynthesis genes detected in all but one isolate and pyoverdine-associated genes present in 6 to 12 isolates, depending on the gene within the operon. Additional variability was observed in adherence and surface-associated virulence loci: type I fimbriae genes were detected in one isolate, type IV pili genes were present in 1 to 10 isolates (depending on the specific gene), and *algP*, encoding a transcriptional regulator of the alginate biosynthesis operon, was detected in 11 isolates. When isolates were stratified by T3SS effector genotype, differences in the total numbers of resistance/virulence genes per isolate were observed (Fig. 4B). *exoU*-positive isolates carried a higher number of acquired resistance genes (range 7–24; mean 16) compared with *exoS*-positive isolates (range 6–12; mean 8). In contrast, *exoU*-positive isolates harboured fewer virulence-associated genes (range 11–28; mean 20) than *exoS*-positive isolates (range 22–39; mean 28). These differences are reported descriptively; given the small number of isolates and the strong relationship between T3SS effector genotype and phylogenetic background, they should not be interpreted as evidence that *exoU* or *exoS* carriage determines overall resistance or pathogenic potential.

**Fig. 4:**
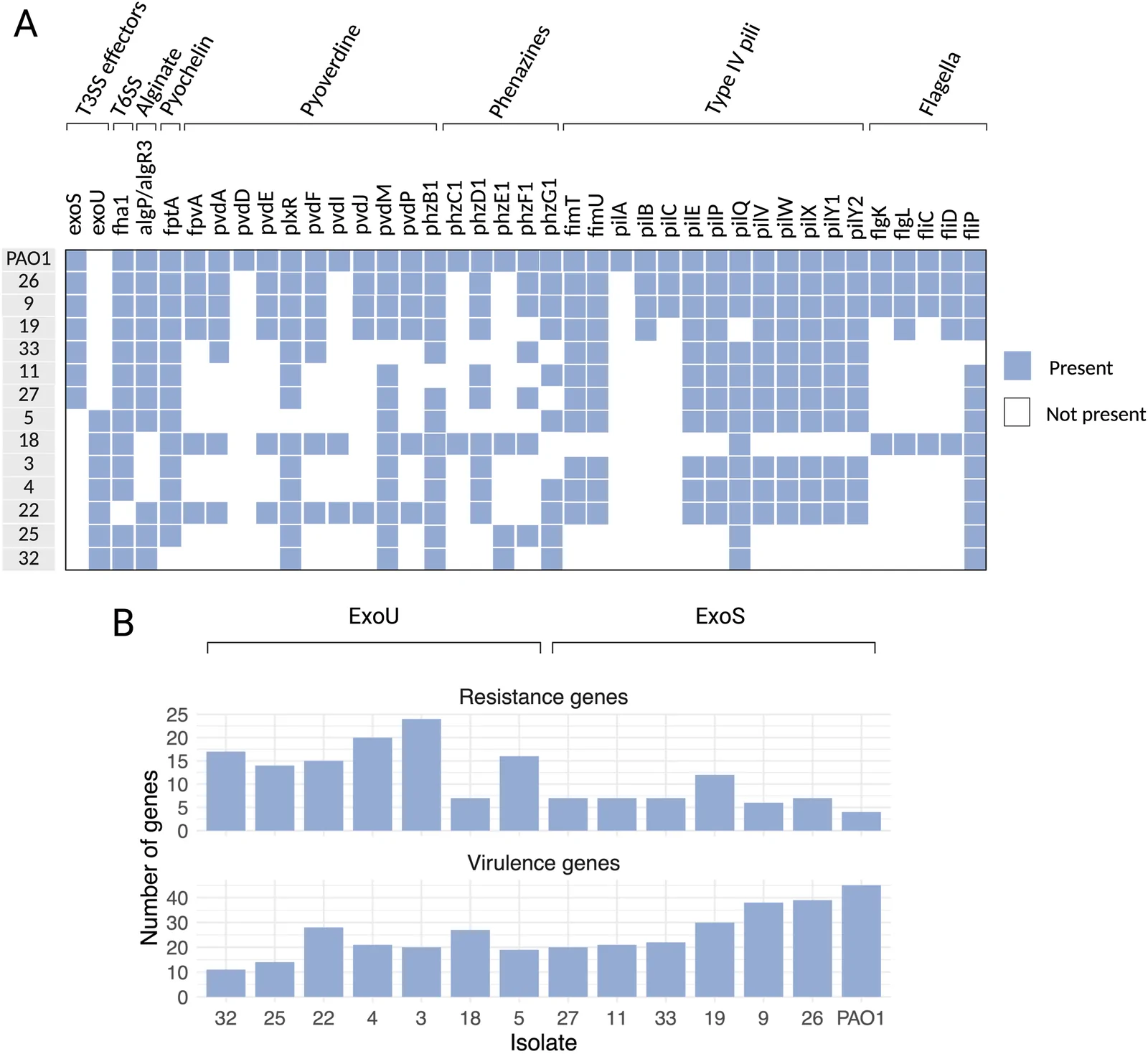
**Virulence gene profiles of*****P. aeruginosa*****isolates from combat-related wounds in Ukraine.** A. Heatmap showing presence (blue) and absence (white) of selected virulence-associated genes across 13 *P. aeruginosa* clinical isolates, with PAO1 included as a reference strain. Genes are grouped by functional category, including type III secretion system (T3SS) effector genes (*exoS*, *exoU*), type VI secretion system–associated genes (T6SS), alginate-associated genes, iron acquisition systems (pyochelin and pyoverdine), phenazine biosynthesis genes, adherence and surface-associated structures (type IV pili), and flagellar genes. Virulence-associated genes were identified and functionally classified according to the Virulence Factor Database (VFDB). Gene presence was defined by ≥ 80% coverage and ≥90% identity and was required for both sequencing duplicates. B. Total number of detected resistance genes and virulence-associated genes per isolate, with isolates grouped by T3SS effector genotype (*exoU*-positive vs *exoS*-positive) and PAO1 shown as a reference.

### 3.5 Heterogeneity in AHL signal production and QS-regulated phenotypes

QS is a central regulatory network in *P. aeruginosa* that coordinates expression of multiple secreted virulence factors and is known to undergo adaptive change during infection [57]. To assess whether virulence-associated phenotypes differed across the clonal backgrounds represented in this collection, we quantified AHL signal production and QS-regulated virulence factor production and compared these data with mutations in QS-associated genes.

Phenotypic analysis revealed substantial variability in AHL signal production and virulence factor production across the isolate collection (Fig. 5A+B). Compared with PAO1, 3-oxo-C12-HSL reporter signal was significantly reduced in 9/13 isolates (isolates 32, 25, 22, 18, 5, 27, 33, 19, and 9; *P* < 0.05 to *P* < 0.0001; Fig. 5A), whereas C4-HSL reporter signal did not differ significantly from PAO1 in the comparisons shown. Rhamnolipid production was significantly reduced in 6/13 isolates (32, 25, 22, 4, 27, and 33; *P* < 0.05; Fig. 5A), while pyocyanin levels varied across isolates without statistically significant differences relative to PAO1. Reduced AHL signal production and QS-regulated phenotypes were therefore observed across several sequence types. Because rhamnolipid production is strongly influenced by growth conditions and medium composition, these differences should be interpreted as comparative production under the standardized experimental conditions used here rather than as condition-independent differences between isolates.

**Fig. 5:**
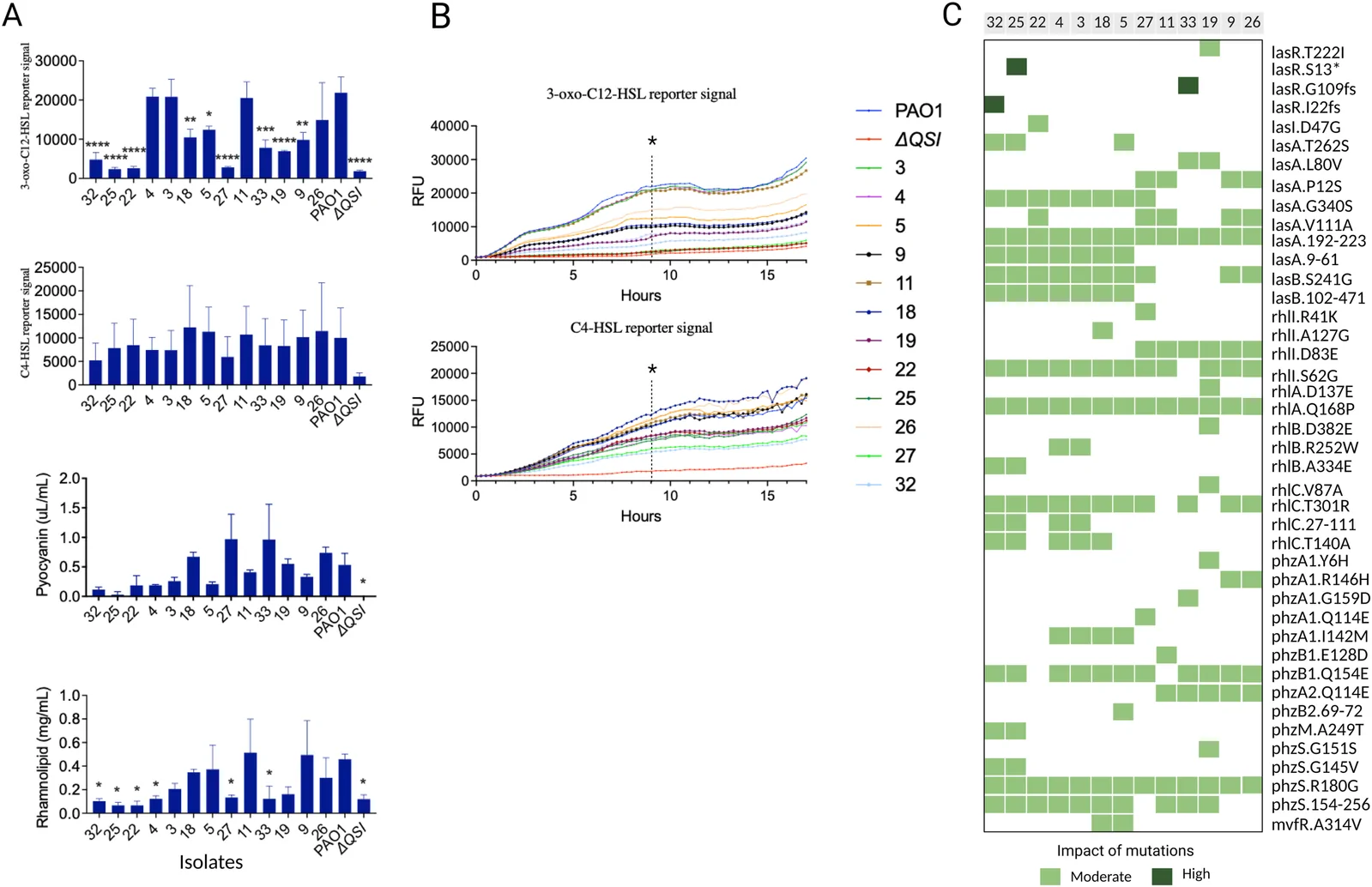
**Phenotypic virulence traits and mutational profiles in QS-associated genes of*****P. aeruginosa*****isolates**. A. Quantitative phenotypic analysis of QS–regulated virulence factors, pyocyanin and rhamnolipids and signal molecules, C4-HSL and 3-oxo-C12-HSL. The results are based on three independent experiments. Error bars represent means ± SDs. *P < 0.05, **P < 0.01, ***P < 0.001, ****P < 0.0001. B. Expression of QS-controlled specific fluorescence (GFP expression/cell density). *Timepoint used for data analysis. C. Presence of moderate- and high-impact mutations in QS–associated genes across isolates. Mutations are grouped by functional pathways, including *las*, *rhl*, *pqs*, and phenazine biosynthesis genes. Each row represents an individual mutation, and columns correspond to isolates.

In three isolates (25, 32, and 33), reduced 3-oxo-C12-HSL production and QS-associated phenotypes coincided with high-impact loss-of-function mutations in *lasR*. In isolate 22, reduced 3-oxo-C12-HSL production coincided with a missense substitution in *lasI* (D47G) affecting a conserved residue reported to be essential for AHL synthase activity [66] consistent with impaired *lasI* function. These isolates belonged to different clonal backgrounds, supporting the interpretation that QS attenuation may arise through distinct genetic routes in different genetic backgrounds rather than reflecting a shared clonal event. In the remaining isolates, no single mutation clearly accounted for the observed phenotypes. Diverse nonsynonymous variants were detected across genes associated with the *las*, *rhl*, and *pqs* QS systems (Fig. 5C–Supplementary Table S12). Although some of these variants may influence protein function, their individual functional consequences were not experimentally determined in this study and they are therefore reported as QS-associated genetic variation rather than as causal determinants of the observed phenotypes.

## 4 Discussion

In this study, we combined WGS with antimicrobial susceptibility testing and virulence phenotyping to characterize the molecular epidemiology, resistance mechanisms, and virulence-associated traits of *P. aeruginosa* isolated from combat-related wounds in Ukraine. The principal findings were: (i) substantial clonal diversity (10 STs among 13 isolates) spanning internationally disseminated high-risk lineages and less well-characterized backgrounds; (ii) a high frequency of MDR/XDR phenotypes with universal non-susceptibility to several first-line antipseudomonal agents and frequent non-susceptibility to last-line β-lactams; (iii) acquired carbapenemases (bla_IMP-1_ and bla_NDM-1_) in all meropenem-non-susceptible isolates alongside substantial chromosomal variation in porin-, efflux-, and β-lactams-response pathways; and (iv) marked heterogeneity in virulence gene content, AHL signal production, and QS-regulated phenotypes, with loss-of-function mutations in *lasR,* or a likely inactivating substitution in *lasI,* in a subset of isolates. Together, these data provide clinically relevant, genomically resolved insight into *P. aeruginosa* from combat-related wounds and support the value of integrated genomic and phenotypic surveillance in conflict-associated healthcare pathways [14], [16].

From a surveillance perspective, our findings suggest that combat-associated *P. aeruginosa* infections are not explained by a single clonal expansion. Instead, we detected multiple internationally disseminated lineages, such as ST235, ST244, ST253, ST773, and ST1047 alongside less well-characterised sequence types ST207, ST270, ST369, ST480 and ST1129. This is consistent with the broader epidemiology of *P. aeruginosa*, where a limited number of globally distributed lineages contribute disproportionately to AMR and outbreaks, while additional backgrounds continue to emerge in specific healthcare networks [17], [18], [20], [67]. ST235 is repeatedly linked to hospital outbreaks and carbapenemase acquisition, and its detection in this collection is consistent with the global dissemination of this high-risk lineage. This mixed population structure is consistent with multiple potential sources of acquisition, including field contamination, initial hospital care, and subsequent referral centres, although the present data cannot distinguish among these possibilities. Although individual pre-hospital antibiotic administration could not be verified in this study, empirical antimicrobial exposure during evacuation and early field care is common in this setting and may influence wound microbiota and early selection pressure. The presence of ST1047 is particularly notable, as genomic studies have linked this lineage to conflict- and displacement-associated healthcare networks and carbapenemase-associated subclonal structure, underscoring the value of lineage-resolved surveillance where inter-facility transfer and cross-border evacuation occur [23].

Phenotypically, all isolates were non-susceptible to ciprofloxacin, ceftazidime, piperacillin–tazobactam, and aztreonam, with 4/13 classified as XDR and 9/13 as MDR. Non-susceptibility to meropenem (4/13) and ceftolozane–tazobactam (5/13) is clinically concerning because these drugs represent key components of contemporary antipseudomonal therapy, especially where treatment options are already constrained. This aligns with the broader AMR observations in which carbapenem resistance is a major driver of mortality and restricted treatment options, and with prioritization frameworks emphasizing the urgency of resistant Gram-negative pathogens [8], [35].

Mechanistically, meropenem non-susceptibility in this collection was restricted to isolates carrying bla_IMP-1_ or bla_NDM-1_, supporting the major role of metallo-β-lactamases in limiting carbapenem activity [68]. The distribution of mutations affecting porins, efflux regulation, and β-lactam response pathways is consistent with established models of resistance in *P. aeruginosa*, in which OprD impairment, AmpC pathway dysregulation, and efflux derepression contribute to multidrug and β-lactam resistance phenotypes [6]. In addition, identification of *rmtB* in ST773 underscores the importance of screening for 16S rRNA methyltransferases, which can confer high-level aminoglycoside resistance and substantially reduce remaining treatment options [31].

Importantly, the number of virulence-associated genes identified in an isolate should not be interpreted as a direct measure of pathogenic potential, which depends on the identity, expression, regulation, and functional activity of individual determinants, as well as on the host and infection environment. Among the variable virulence determinants identified in our collection, the T3SS effectors *exoU* and *exoS* are particularly relevant because they mediate distinct interactions with host cells [34], [69]. ExoU is a potent phospholipase associated with rapid cytotoxicity and tissue damage, whereas ExoS has GTPase-activating and ADP-ribosyltransferase activities that interfere with host-cell signalling and cytoskeletal functions [34]. The mutually exclusive distribution of *exoU* and *exoS* across our isolates is consistent with the broader population structure of *P. aeruginosa*, in which *exoS* is strongly associated with the PAO1-like major lineage and *exoU* with the PA14-like lineage [32], [33].

In parallel, production of 3-oxo-C12-HSL and several QS-regulated phenotypes were reduced in multiple isolates relative to PAO1. In a subset of isolates, these phenotypes coincided with loss-of-function mutations in *lasR* and a potentially inactivating substitution in *lasI*, whereas in others no single explanatory mutation was identified. Variation in *lasR* is common among *P. aeruginosa* isolates and is not restricted to a particular infection type or clinical setting [70]. Thus, the *lasR* variants observed in our combat-wound isolates are consistent with the broader diversity of this regulator and should not necessarily be interpreted as specific adaptations to combat-associated wound infection. The additional variation observed across *rhl*, *pqs*, and downstream QS-associated pathways further suggests that differences in QS-related phenotypes may arise through multiple genetic routes in different clonal backgrounds. These observations emphasize that virulence-associated phenotypes cannot be inferred from resistance profile or genomic background alone and support the value of combining genomic profiling with phenotypic assays in this setting.

Several limitations of this study should be acknowledged. The collection comprised 13 isolates from 13 distinct patients, which limits statistical power for genotype–phenotype comparisons and precludes definitive conclusions about clonal expansion, ST- or genetic background-associated traits, or population-level epidemiology. Clinical metadata, including patient outcomes, infection severity, antibiotic treatment history, and transmission events, were not available, restricting the translational and infection-control conclusions that can be drawn. Despite these limitations, the integrated approach combining WGS, multi-tool resistance profiling, and quantitative virulence phenotyping provides a methodological framework applicable to future, larger surveillance studies in conflict-associated healthcare settings.

## 5 Conclusion

In conclusion, combat-wound *P. aeruginosa* isolates from Ukraine comprised diverse STs and genomic backgrounds with frequent MDR phenotypes and clinically important acquired resistance determinants, including *bla*IMP-1 and *bla*NDM-1. Virulence gene repertoires, AHL-signal production, and QS-regulated phenotypes were also heterogeneous across the collection. These findings support integrating rapid antimicrobial susceptibility testing with lineage-resolved genomic surveillance to inform antimicrobial management, infection prevention and control, and cross-border surveillance in conflict-associated healthcare pathways.

## CRediT authorship contribution statement

**Oana Palasca:** Writing – review & editing, Writing – original draft, Validation, Investigation, Formal analysis, Data curation. **Caroline Hvidberg Schwartz:** Writing – review & editing, Writing – original draft, Methodology, Investigation, Data curation. **Rasmus Kristensen:** Writing – review & editing, Methodology, Investigation. **Iryna Koshova:** Writing – review & editing, Methodology, Investigation. **Bohdan Murashevych:** Writing – review & editing, Methodology, Investigation, Funding acquisition. **Trine Rolighed Thomsen:** Writing – review & editing, Investigation, Funding acquisition, Conceptualization. **Frederik Boëtius Hertz:** Writing – review & editing, Validation, Methodology, Investigation. **Thomas Bjarnsholt:** Writing – review & editing, Validation, Investigation. **Tim Holm Jakobsen:** Writing – review & editing, Writing – original draft, Validation, Supervision, Project administration, Methodology, Investigation, Funding acquisition, Formal analysis, Data curation, Conceptualization.

## Ethical approval statement

Formal ethical approval was not required for this study. All bacterial isolates were collected as part of routine clinical microbiological practice in Ukraine. No identifiable patient data were obtained, and all microbiological data were processed and analysed anonymously. Written or verbal informed consent was therefore not required.

## Availability of data and materials

Raw sequencing reads and genome assemblies have been deposited in NCBI under BioProject PRJNA1473102. SRA accessions: SRR38939230–SRR38939242. BioSample accessions: SAMN60549715–SAMN60549727. Genome assemblies: GenBank accessions *JBYWLD000000000–JBYWLP000000000.*

Reviewer access link: https://dataview.ncbi.nlm.nih.gov/object/PRJNA1473102?reviewer=5og419crvsvfq35bk194trni0o.

## Funding

This work was supported by the National Defence Technology Centre (NFC) [25204 and 24025] to THJ and TRT and by the Ministry of Health of Ukraine [0126U001725] to BM.

## Declaration of competing interest

The authors declare that they have no known competing financial interests or personal relationships that could have appeared to influence the work reported in this paper.
