# Supplementary material for: Molecular epidemiology and virulence profiles of MDR and XDR Pseudomonas aeruginosa from combat-related wounds in Ukraine
Source: New Microbes New Infect. 2026 Oct 30;74:101880. doi: 10.1016/j.nmni.2026.101880 (PMC13756339; doi:10.1016/j.nmni.2026.101880)
Supplement: Supplementary file 1 [file mmc1.docx]

**Table S1: Strain collection used in the study**

| Strain | Species | Source/reference | NCBI accession number |
| --- | --- | --- | --- |
| PAO1 | *P. aeruginosa* | Strain collection, CBC, Copenhagen, Denmark | AE004091.2 |
| ATCC 25922 | *E. coli* | Strain collection,  Rigshospitalet, Copenhagen, Denmark | CP009072.1 |
| Carbapenemase-positive isolate | *K. pneumoniae* | This study | - |
| MH205 | *E. coli* | Garde C. et al. 2010^1^ | - |
| MH155 | *E. coli* | Hentzer M. et al. 2002^2^ | - |
| *∆QSI* | *P. aeruginosa* | M. E. Skindersoe et al. 2008^3^ | - |
| Clinical isolate #3 | *P. aeruginosa* | This study | - |
| Clinical isolate #4 | *P. aeruginosa* | This study | - |
| Clinical isolate #5 | *P. aeruginosa* | This study | - |
| Clinical isolate #9 | *P. aeruginosa* | This study | - |
| Clinical isolate #11 | *P. aeruginosa* | This study | - |
| Clinical isolate #18 | *P. aeruginosa* | This study | - |
| Clinical isolate #19 | *P. aeruginosa* | This study | - |
| Clinical isolate #22 | *P. aeruginosa* | This study | - |
| Clinical isolate #25 | *P. aeruginosa* | This study | - |
| Clinical isolate #26 | *P. aeruginosa* | This study | - |
| Clinical isolate #27 | *P. aeruginosa* | This study | - |
| Clinical isolate #32 | *P. aeruginosa* | This study | - |
| Clinical isolate #33 | *P. aeruginosa* | This study | - |

**References**

1.	Garde C, Bjarnsholt T, Givskov M, Jakobsen TH, Hentzer M, Claussen A, et al. Quorum sensing regulation in Aeromonas hydrophila. J Mol Biol. 2010;396(4):849-57.

2.	Hentzer M, Riedel K, Rasmussen TB, Heydorn A, Andersen JB, Parsek MR, et al. Inhibition of quorum sensing in Pseudomonas aeruginosa biofilm bacteria by a halogenated furanone compound. Microbiology (Reading). 2002;148(Pt 1):87-102.

3.	Skindersoe ME, Alhede M, Phipps R, Yang L, Jensen PO, Rasmussen TB, et al. Effects of antibiotics on quorum sensing in Pseudomonas aeruginosa. Antimicrob Agents Chemother. 2008;52(10):3648-63.
